# Imaging-Based Phenotyping in Bicuspid Aortic Valve and Associated Aortopathy: A Retrospective Single-Center Study

**DOI:** 10.3390/medicina62040790

**Published:** 2026-04-20

**Authors:** Laurynas Šarkinas, Nomeda Rima Valevičienė, Sigita Glaveckaitė, Darius Palionis

**Affiliations:** 1 Faculty of Medicine, Vilnius University, LT-03101 Vilnius, Lithuania; 2 Department of Radiology, Nuclear Medicine and Medical Physics, Institute of Biomedical Sciences, Vilnius University, LT-08661 Vilnius, Lithuania; 3 Clinic of Heart and Vascular Diseases, Institute of Clinical Medicine, Faculty of Medicine, Vilnius University, LT-08661 Vilnius, Lithuania

**Keywords:** bicuspid aortic valve, aortopathy, multimodality imaging, magnetic resonance imaging, echocardiography

## Abstract

*Background and Objectives*: Bicuspid aortic valve (BAV) is a common congenital cardiac abnormality frequently associated with aortopathy and progressive aortic dilatation. The 2021 International consensus statement introduced standardized phenotypic definitions for BAV and BAV-associated aortopathy. The aim of this study was to determine the distribution of BAV and BAV-associated aortopathy phenotypes according to the 2021 consensus statement and to assess statistical differences between imaging modalities (magnetic resonance imaging (MRI) and transthoracic echocardiography (TTE)) in aortic diameter measurements. *Materials and Methods*: This single-center retrospective study included 47 patients with BAV between 2014 and 2018. Aortic root and ascending aorta diameters were measured using MRI white-blood and black-blood sequences and TTE. BAV morphology and aortopathy phenotypes were classified according to the 2021 International consensus recommendations, and agreement between modalities was assessed using correlation and Bland–Altman analysis. *Results*: The study population had a mean age of 30 ± 11.05 years, with a male-to-female ratio of approximately 3.3:1. The fused BAV phenotype was the most prevalent (53.2%), followed by the partial-fusion (38.3%) and two-sinus (8.5%) phenotypes (*p* < 0.001). Aortopathy phenotypes assessed by MRI and TTE demonstrated similar distribution patterns, with the extended phenotype being the most frequent, followed by ascending and root phenotypes, with no significant difference between modalities (*p* = 0.74). Strong correlations were observed across all imaging techniques for both ascending aorta and aortic root measurements; however, small but statistically significant differences were identified between selected modality pairs—a mean difference of 1.59 mm between MRI black-blood and TTE ascending aorta diameters and 1.26 mm between MRI white-blood and TTE aortic root diameters. *Conclusions*: Multimodality imaging demonstrates strong agreement in the assessment of aortic diameters and yields comparable aortopathy phenotype distributions when applying the 2021 International Consensus Classification. Nevertheless, small systematic measurement differences between MRI and TTE may be clinically relevant in patients approaching diagnostic or therapeutic thresholds, highlighting the importance of consistent imaging methodology in longitudinal follow-up of BAV-associated aortopathy.

## 1. Introduction

Bicuspid aortic valve (BAV) is one of the most common congenital cardiac malformations, occurring in approximately 1–2% of the general population, with a male-to-female ratio of 3:1 [1]. BAV may occur as an isolated valvular abnormality or in association with aortopathy, which involves dilatation of the proximal aorta, including the aortic root, ascending aorta, or both. The latter condition is clinically significant, as it may lead to aneurysm formation, aortic dissection, or rupture. Although aortic stenosis, aortic regurgitation, and endocarditis are more frequent complications, aortic dilatation remains highly prevalent, occurring in up to 50% of cases [2]. Moreover, BAV-related aortopathy is recognized as a heterogeneous condition with variable phenotypic expression, which may partly explain differences in clinical outcomes [2,3,4].

Until the release of the 2021 International consensus statement, there had been no unified nomenclature or classification system for BAV, resulting in considerable heterogeneity and confusion across studies [5]. The consensus introduced a standardized classification of BAV into three major morphological types (fused, two-sinus, and partial-fusion) and three aortopathy phenotypes (root, ascending, and extended). This classification enabled more consistent assessment across diagnostic imaging modalities.

Nevertheless, registry data suggest that the prevalence of different BAV and aorto-pathy phenotypes varies substantially across populations [4,6,7], highlighting the importance of standardized imaging evaluation. Comparative data on measurement accuracy between magnetic resonance imaging (MRI) and transthoracic echocardiography (TTE) in BAV-related aortopathy remain limited. This study aimed to evaluate and compare aortic root and ascending aorta diameter measurements in BAV patients using MRI and TTE and to determine the distribution of BAV and aortopathy phenotypes in the cohort.

## 2. Materials and Methods

We retrospectively analyzed 47 patients with BAV who were identified from the registry of Vilnius University Hospital Santaros Clinics from 2014 to 2018. The study was conducted in accordance with the recommendations of the Regional Biomedical Research Ethics Committee with permission No. 158200-13-576-178/12 February 2013 (Supplement No. 158200-576-PP1-14). Patients were included if they had a confirmed diagnosis of BAV established by imaging (MRI or TTE). Basic demographic and anthropometric parameters, including age, sex, body weight, height, and body surface area (BSA), were collected for all participants. All patient data were anonymized, and informed consent was obtained prior to inclusion.

**Figure 1:**
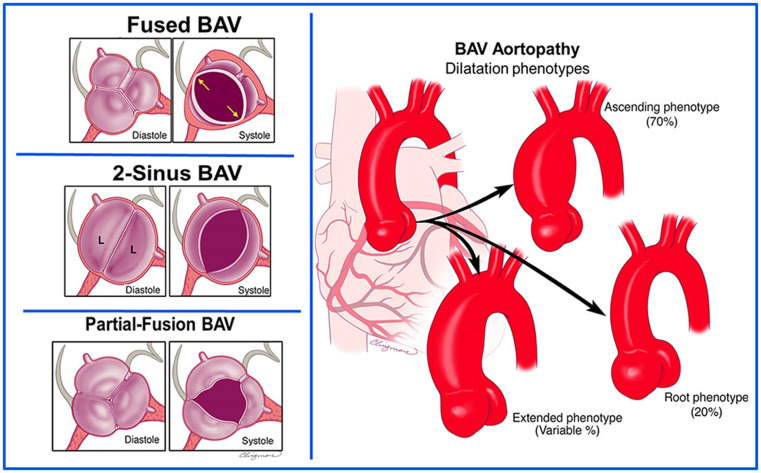
Morphological phenotypes of bicuspid aortic valve and BAV-associated aortopathy. Schematic illustration of bicuspid aortic valve (BAV) morphological phenotypes (on the (**left**)), including fused BAV, two-sinus BAV, and partial-fusion BAV and schematic representation of BAV-related aortic dilatation phenotypes (on the (**right**)), including ascending, root, and extended phenotypes. Adapted from Michelena et al., International consensus statement on nomenclature and classification of the congenital bicuspid aortic valve and its aortopathy, European Journal of Cardio-Thoracic Surgery, 2021 [5]. L: lateral cusp.

BAV morphology and associated aortopathy phenotypes were classified according to the 2021 International consensus statement on nomenclature and classification of the congenital bicuspid aortic valve and BAV aortopathy [5]. Three BAV types (fused, two-sinus, and partial-fusion) and three aortopathy phenotypes (root, ascending, and extended) were systematically assessed (Figure 1).

**Figure 2:**
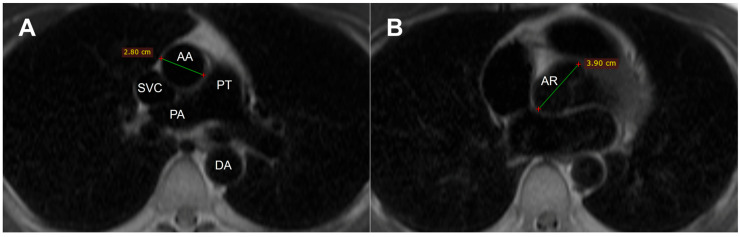
Aortic diameters were measured on axial magnetic resonance images at standardized anatomical landmarks using white-blood and black-blood MRI sequences. (**A**) Ascending aorta (AA) diameter measured at the level of the main pulmonary artery. (**B**) Aortic root (AR) diameter measured at the level of the sinuses of Valsalva. The green line indicates the measured diameter. AA: Ascending aorta; AR: Aortic root; SVC: Superior vena cava; PT: Pulmonary trunk; PA: Pulmonary artery; DA: Descending aorta.

Radiological data were analyzed using RadiAnt DICOM Viewer software (version 2025.1; Medixant, Poznan, Poland). Each patient underwent cardiac MRI with white-blood (WB) and black-blood (BB) sequences as well as TTE. BAV type was determined by analyzing short-axis aortic valve imaging with MRI or TTE. On TTE aortic root and ascending aorta were measured in the parasternal long-axis view from the leading edge to the leading edge of the other wall in diastole; aortic valve anatomy was assessed in parasternal short axis view by zooming aortic valve and choosing proper angulation of the transducer. Aortic root (AR) diameter was measured in the phase where the aortic sinuses appeared widest, typically in the short-axis view during diastole from the inner to the inner edge. Ascending aortic diameter (AA) was measured at the level of the right pulmonary artery from the inner to the inner edge by using double angulation technique [8] (Figure 2). All measurements were performed by one experienced physician, specialized in cardiovascular imaging.

To adjust aortic dimensions for body size, z-scores were calculated using the Boston Children’s Hospital calculator, with a z-score >+2 considered indicative of pathological dilatation [9]. Statistical analysis was performed using Microsoft Excel (Microsoft Corporation, Redmond, WA, USA), RStudio (version 2024.12.1; Posit Software, Boston, MA, USA) with the Rcmdr package (version 4.4.3), and MedCalc (version 23.4.0; MedCalc Software Ltd., Ostend, Belgium). A *p*-value of <0.05 was considered statistically significant. Data distribution was evaluated using the Shapiro–Wilk test. Distribution of BAV phenotypes was assessed using frequency tables and the chi-square goodness-of-fit test. Distribution of aortopathy phenotypes across imaging modalities was compared using the chi-square test of independence or Fisher’s exact test, as appropriate. Correlations were assessed by Pearson’s or Spearman’s coefficient, depending on data normality. Mean differences between ascending aorta and aortic root diameters measured using different imaging modalities (MRI BB, MRI WB, and TTE) were evaluated using paired statistical analyses, resulting in six modality-specific comparisons. Normality of paired differences was assessed prior to testing. For normally distributed paired data, paired *t*-tests were applied. Outlier assessment was performed using boxplot-based criteria, and where necessary, exclusion of a single observation was undertaken to satisfy normality assumptions. For paired data that did not follow a normal distribution, the Wilcoxon signed-rank test was used. Agreement between imaging modalities was further evaluated using Bland–Altman analysis.

## 3. Results

### 3.1. Baseline Characteristics

**Figure 3:**
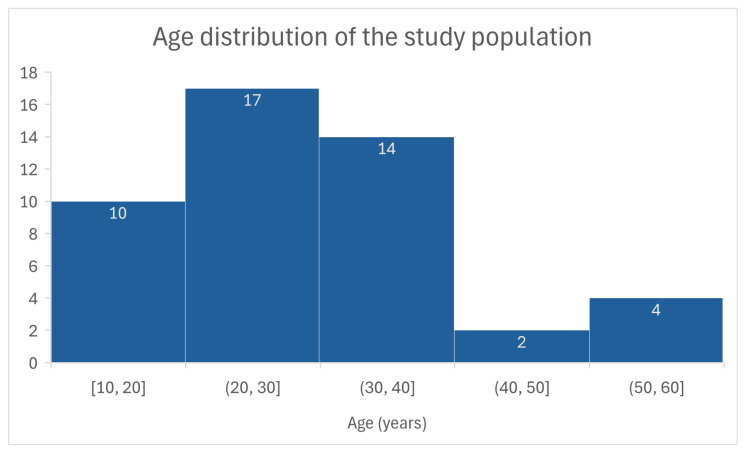
Histogram illustrating the age distribution of the study population using 10-year intervals. Numbers above bars indicate the number of patients in each age group.

Our study included 47 patients from the registry of Vilnius University Hospital Santaros Clinics with BAV. Of these 47 patients, 36 (76.6%) were male and 11 (23.4%) were female, corresponding to a male-to-female ratio of approximately 3.3:1. Although the mean age of the cohort was 30 ± 11.05 years, the age distribution was non-normal according to the Shapiro–Wilk test (*p* < 0.05). Therefore, the median age (29 years) provides a more appropriate measure of central tendency for this sample. Figure 3 illustrates the age distribution of the study cohort. In addition, the mean weight of a patient was 74.6 ± 14.4 kg and height was 176.8 ± 11.7 cm, with an average BSA of 1.90 ± 0.23 m^2^. Additional clinical characteristics of the study cohort, including aortic diameters and corresponding z-scores, are presented in Table 1.

### 3.2. BAV Phenotype Distribution

**Figure 4:**
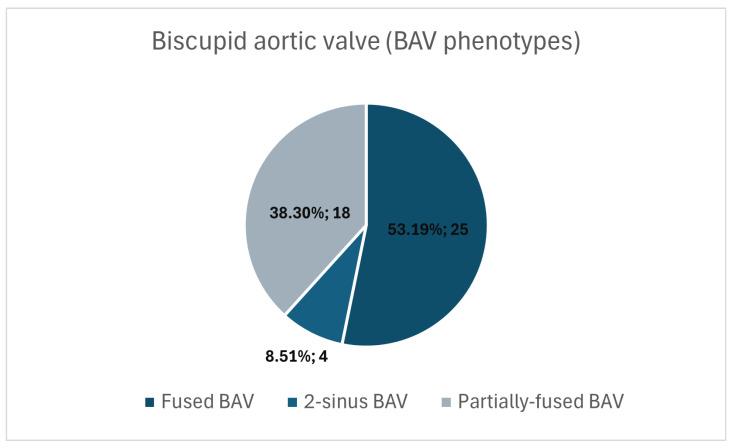
Pie chart illustrating the distribution of bicuspid aortic valve morphological phenotypes in the study cohort. Values represent absolute counts and corresponding percentages.

In our cohort (n = 47), the fused BAV phenotype was predominant, accounting for 53.2% (25 cases), followed by the partial-fusion phenotype with 38.3% (18 cases) and the two-sinus phenotype 8.5% (4 cases) (Figure 4). The pattern of BAV phenotype distribution in our cohort was similar to that reported in the Polish RE-BAV Registry and Italian cohort [6,10], with the fused phenotype representing the most common subtype.

### 3.3. Aortopathy Phenotype Distribution Across Imaging Modalities

#### 3.3.1. MRI Black-Blood Sequences

**Figure 5:**
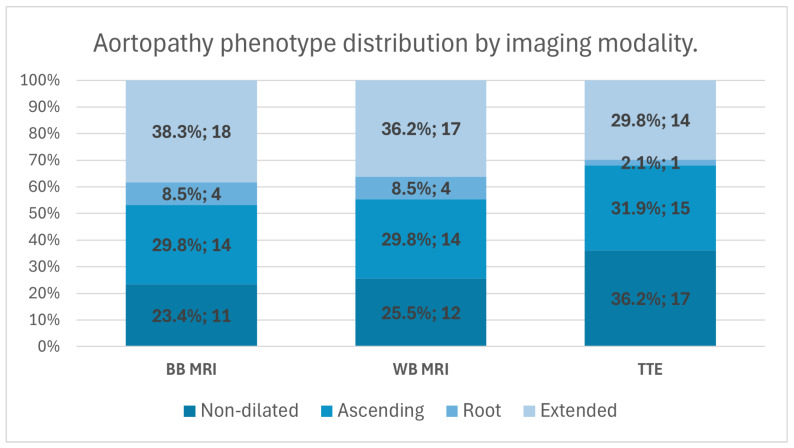
Stacked bar chart illustrates the distribution of BAV-associated aortopathy phenotypes assessed using MRI black-blood (BB), MRI white-blood (WB), and transthoracic echocardiography (TTE). Bars represent relative proportions of non-dilated, ascending, root, and extended aortopathy phenotypes. Values indicate absolute counts with corresponding percentages.

Using MRI black-blood imaging, aortic dilatation defined by z-scores was present in 76.6% of patients. Among those with dilatation, the extended phenotype was the most prevalent (38.3%), followed by the ascending phenotype (29.8%) and the root phenotype (8.5%) (Figure 5).

#### 3.3.2. MRI White-Blood Sequences

Assessment with MRI white-blood sequences demonstrated aortic dilatation in 74.4% of cases. The extended phenotype remained the most frequent pattern (36.2%), while the ascending and root phenotypes accounted for 29.8% and 8.5% of cases, respectively.

#### 3.3.3. Transthoracic Echocardiography

Using transthoracic echocardiography, aortic dilatation according to the z-score was identified in 63.8% of patients. Within this group, the ascending phenotype was the most common (31.9%), followed by the extended phenotype (29.8%), whereas the root phenotype was rarely observed (2.1%).

### 3.4. Data Distribution and Descriptive Statistics

Descriptive statistical analysis revealed that among the demographic variables, age was not normally distributed, as assessed by the Shapiro–Wilk test (*p* < 0.05). In addition, non-normal distributions were observed for ascending aorta diameters measured using MRI black-blood and white-blood sequences, as well as for BB ascending aorta and WB aortic root z-scores (Table 1). All other measured variables followed a normal distribution, and therefore parametric statistical methods were applied accordingly.

### 3.5. Correlation Between Imaging Modalities

Correlation analysis demonstrated strong associations between all measured diameters of the ascending aorta and the aortic sinuses across imaging sequences (WB, BB sequences and TTE) (Table 2). Depending on data distribution, either Spearman’s rank correlation or Pearson’s correlation coefficient was applied. The weakest observed correlation was 0.793, still indicating a strong relationship, whereas the strongest correlations were identified between ascending aorta MRI WB and BB diameter measurements (ρ = 0.966, Spearman) and between aortic root MRI WB and BB diameter measurements (r = 0.861, Pearson).

### 3.6. Measurement Differences Between Imaging Methods

**Figure 6:**
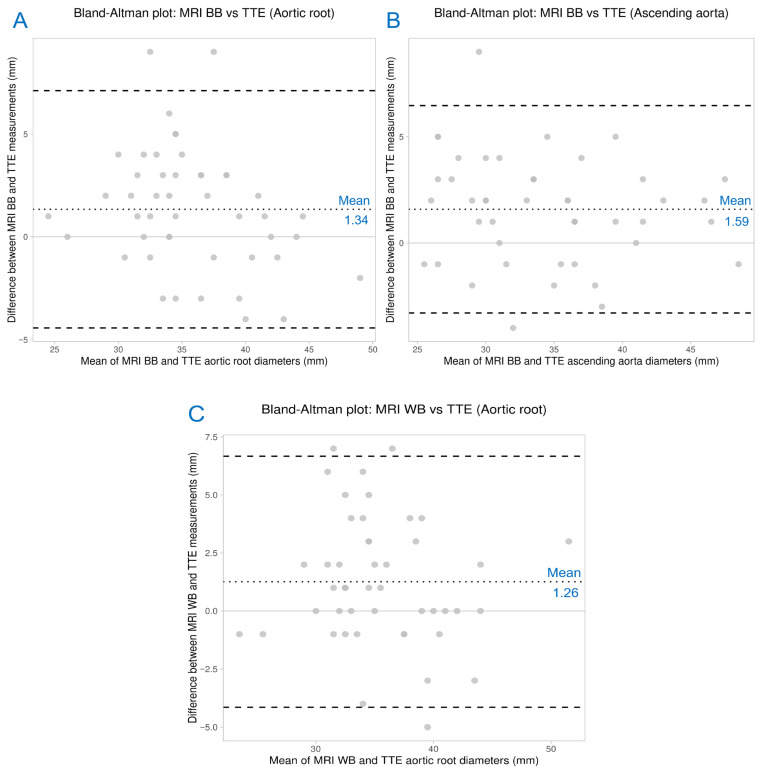
Bland–Altman plots illustrating agreement between magnetic resonance imaging (MRI) and transthoracic echocardiography (TTE) for aortic diameter measurements. (**A**) Comparison of MRI black-blood (BB) and TTE aortic root diameters. (**B**) Comparison of MRI black-blood (BB) and TTE ascending aorta diameters. (**C**) Comparison of MRI white-blood (WB) and TTE aortic root diameters. The dotted line represents the mean difference (bias), the dashed lines indicate the 95% limits of agreement, and the solid horizontal line represents zero difference.

Among the six modality-specific comparisons, statistically significant mean differences were identified for selected aortic root diameter measurements. A mean difference of 1.34 mm was observed between MRI black-blood and echocardiographic aortic root diameters using paired *t*-testing. After exclusion of one outlier to achieve normality, a mean difference of 1.59 mm was found between MRI black-blood and echocardiographic ascending aorta diameters. Similarly, following removal of a single outlier, comparison between MRI white-blood and echocardiographic aortic root diameters yielded a mean difference of 1.26 mm. These findings were illustrated using three Bland–Altman plots (Figure 6). No statistically significant differences were observed in the remaining modality pairs. Comparisons analyzed using the Wilcoxon signed-rank test did not demonstrate clinically meaningful differences and are therefore not reported in detail.

## 4. Discussion

When comparing our cohort’s BAV phenotype distribution (53.2% fused, 38.3% partial-fusion, and 8.5% two-sinus types) with that reported in the Polish RE-BAV Registry and Italian cohort [6,10], a broadly similar distribution pattern was observed, with the fused phenotype representing the most prevalent subtype in all three cohorts. In the Polish RE-BAV Registry, fused BAV accounted for approximately 79.4% of cases, followed by the two-sinus (15.8%) and partial-fusion (4.8%) phenotypes, whereas in the Italian cohort, the fused phenotype similarly predominated (81.1%), with lower relative proportions of two-sinus (13.6%) and partial-fusion (5.3%) types. However, differences in the relative proportions of the partial-fusion and two-sinus phenotypes were noted. Such variation may be partly explained by the substantially smaller sample size of our study and potential regional and genetic differences [11]. Importantly, BAV morphological phenotyping is inherently subjective, as it relies on the assessment of multiple valve-related features, including cusp fusion patterns, raphé presence, and commissural orientation [12]. These elements may be interpreted differently depending on imaging quality, observer experience, and analytical approach, thereby introducing interobserver variability—an effect that may be particularly pronounced in smaller cohorts [13].

In contrast to BAV morphology, aortopathy phenotype classification is based on measured diameters of the aortic sinuses and the ascending aorta at predefined anatomical landmarks, representing a more objective and reproducible approach [14]. This reliance on quantitative measurements not only facilitates robust inter-study comparisons and reduces susceptibility to observer-related variability, but also aligns more closely with clinically relevant decision-making, which is largely guided by absolute aortic dimensions [15]. Using transthoracic echocardiography and MRI white-blood and black-blood sequences, the distribution pattern of aortopathy phenotypes in our cohort closely mirrored that reported in the Polish RE-BAV Registry, with the extended phenotype being the most prevalent, followed by the ascending and root phenotypes [6]. These results provide empirical support for the practical applicability of the 2021 International Consensus Classification, demonstrating that measurement-based aortopathy phenotyping yields consistent and comparable results across imaging modalities and cohorts. Collectively, these findings underscore the importance of measurement-based classification strategies for reliable phenotypic characterization and cross-cohort comparability.

An additional methodological consideration relates to differences in aortopathy assessment criteria between studies. In the present study, aortic dilatation was defined using body size-adjusted z-scores, with values exceeding +2 considered pathological [16]. In contrast, the RE-BAV Registry applied fixed absolute diameter thresholds in men and women. Although both approaches are widely accepted, z-score-based assessment may be more sensitive in younger or smaller-bodied individuals, potentially influencing phenotype classification [17]. This methodological discrepancy should be taken into account when interpreting inter-study comparisons, even though overall aortopathy phenotype distributions assessed using measurement-based methods remained comparable between cohorts.

Although the observed differences in ascending aorta and aortic root diameter measurements between MRI black-blood, white-blood, and transthoracic echocardiography were small, they remain clinically relevant. Even minor systematic differences across imaging modalities may influence longitudinal follow-up, risk stratification, and the timing of clinical decision-making, particularly in patients with measurements approaching established thresholds for aortic dilatation or surgical intervention [14,18]. For instance, a systematic measurement difference of approximately 1–1.5 mm, although seemingly small, may affect whether a patient with borderline aortic root or ascending aorta dilatation meets guideline-recommended criteria for surgical consideration (≥55 mm), as outlined in the 2024 ESC guidelines for the management of peripheral arterial and aortic diseases [14]. Differences between modalities may reflect distinct image acquisition principles, spatial resolution, and edge-definition characteristics, with MRI sequences and echocardiography varying in their depiction of the aortic wall and lumen boundaries [19,20]. Consequently, consistent use of the same imaging modality and measurement technique is essential for reliable serial assessment. Our findings highlight that while multimodality imaging provides complementary information, awareness of modality-specific measurement characteristics is crucial to avoid misinterpretation of small but systematic diameter differences. It should also be noted that population-based data on the distribution of BAV morphological subtypes and associated aortopathy phenotypes according to the 2021 International Consensus Classification remain limited [5]. To date, only a small number of registries and cohort studies have systematically applied this novel classification framework [6,10]. Therefore, the present study contributes additional data to a still emerging body of the literature and provides insight into phenotype distributions within a relatively young European cohort assessed using contemporary imaging criteria.

From a clinical and imaging perspective, our findings support the use of standardized, measurement-based approaches for aortopathy phenotyping in patients with BAV. Reliance on direct aortic diameter measurements at predefined anatomical landmarks appears to provide reproducible and comparable results. Incorporating quantitative criteria into routine imaging evaluation may therefore improve phenotypic classification, facilitate inter-study comparisons, and enhance longitudinal follow-up of patients with BAV-associated aortopathy.

## 5. Limitations

Several limitations should be acknowledged. First, the relatively small sample size (n = 47) may limit statistical power and contribute to observed differences in BAV phenotype distribution. Nevertheless, it represents one of the few eastern European cohorts reported in the literature to date. Second, the retrospective design and use of registry data may introduce selection bias. Having said that, the use of standardized classification criteria and multimodality imaging strengthens the validity of the present findings. The measurements were carried out by a single expert, which is a limitation. However, the expert was highly specialized and had extensive experience, which increases the reliability of their measurements.

## 6. Conclusions

In this single-center cohort, the fused BAV phenotype was the most prevalent, followed by the partial-fusion and two-sinus phenotypes. Regarding BAV-associated aorto-pathy, dilatation of the thoracic aorta was frequently observed, with the extended and ascending phenotypes being the most prevalent, whereas the root phenotype was considerably less common. Statistically significant, albeit modest, discrepancies exist between ascending aorta and aortic root diameters measured across MRI and TTE. While minimal, these variations can impact clinical management, especially when tracking longitudinal progression or approaching intervention thresholds. Consequently, standardized measurement protocols and consistent imaging techniques are essential for accurate phenotypic classification and optimized decision-making in BAV patients.

## Figures and Tables

**Table 1:** Baseline characteristics and aortic measurements of the study population.

Variable	Mean ± SD	Median (IQR)	Min–Max	Shapiro–Wilk *p*-Value
Demographic Variables				
Age (years) BSA (m^2^)	30.38 ± 11.05	29.00 (21.50–38.50)	13.00–56.00	0.047 ^1^
	1.90 ± 0.23	1.90 (1.74–2.05)	1.50–2.43	0.352
Sex ^2^				
Males (%)	36 (76.6%)			
Females (%)	11 (23.4%)			
Ascending Aorta (AA) Diameter				
BB (mm) WB (mm)	35.47 ± 6.37	35.00 (30.50–38.00)	25–50	0.033 ^1^
	34.77 ± 6.44	34.00 (29.00–38.00)	25–50	0.026 ^1^
TTE (mm)	33.49 ± 6.40	32.00 (29.00–37.00)	24–49	0.091
Ascending Aorta (AA) Z-score				
BB WB TTE	3.45 ± 2.31	2.77 (1.80–5.27)	0.20–8.97	0.011 ^1^
	3.19 ± 2.29	2.90 (1.37–4.64)	−0.37–9.16	0.068
	2.72 ± 2.25	2.35 (1.05–4.26)	−1.35–8.00	0.235
Aortic Root (AR) Diameter				
BB (mm)	36.36 ± 4.70	37.00 (33.00–40.00)	25–48	0.703
WB (mm)	35.98 ± 5.11	36.00 (33.00–39.50)	23–53	0.088
TTE (mm)	35.02 ± 5.50	34.00 (31.50–38.50)	24–50	0.184
Aortic Root (AR) Z-score				
BB	1.84 ± 1.76	1.87 (0.73–3.10)	−1.63–6.81	0.705
WB	1.70 ± 2.00	1.43 (0.34–2.73)	−1.63–8.72	0.022 ^1^
TTE	1.35 ± 1.97	1.18 (−0.04–2.55)	−2.40–7.57	0.202

^1^ Variables not following a normal distribution, as assessed by the Shapiro–Wilk test (*p* < 0.05), are better represented by their median values. ^2^ categorical variables (e.g., sex) are presented as number (percentage). Abbreviations: SD—standard deviation; AA—ascending aorta; AR—aortic root; BB—black-blood MRI; WB—white-blood MRI; TTE—transthoracic echocardiography; BSA—body surface area.

**Table 2:** Correlation of ascending aorta and aortic root diameter measurements across imaging modalities.

AA Correlation Coefficient ^1^	MRI BB	MRI WB	TTE
MRI BB	1	0.966	0.855
MRI WB	0.966	1	0.835
TTE	0.855	0.835	1
**AR Diameter Correlation Coefficient** **^2^**	**MRI BB**	**MRI WB**	**TTE**
MRI BB	1	0.861	0.845
MRI WB	0.861	1	0.793
TTE	0.845	0.793	1

^1^ Spearman’s rank correlation coefficient (ρ) was used for AA measurements due to non-normal data distribution. ^2^ Pearson’s correlation coefficient (r) was applied for AR measurements, which followed a normal distribution. Abbreviations: AA—ascending aorta; AR—aortic root; BB—black-blood MRI; WB—white-blood MRI; TTE—transthoracic echocardiography.

## Data Availability

The data presented in this study are available on request from the corresponding author.

## Abbreviations

AA	Ascending aorta
ACC	American College of Cardiology
AHA	American Heart Association
AR	Aortic root
BAV	Bicuspid aortic valve
BB	Black-blood magnetic resonance imaging
BSA	Body surface area
ESC	European Society of Cardiology
IQR	Interquartile range
MRI	Magnetic resonance imaging
RE-BAV	Registry of Bicuspid Aortic Valves
SD	Standard deviation
TTE	Transthoracic echocardiography
WB	White-blood magnetic resonance imaging
